# Contractile Activity Is Necessary to Trigger Intermittent Hypobaric Hypoxia-Induced Fiber Size and Vascular Adaptations in Skeletal Muscle

**DOI:** 10.3389/fphys.2018.00481

**Published:** 2018-05-04

**Authors:** David Rizo-Roca, Jèssica B. Bonet, Büsra Ínal, Juan Gabriel Ríos-Kristjánsson, Teresa Pagès, Ginés Viscor, Joan R. Torrella

**Affiliations:** ^1^Unitat de Fisiologia, Departament de Biologia Cel⋅lular, Fisiologia i Immunologia, Facultat de Biologia, Universitat de Barcelona, Barcelona, Spain; ^2^LaMetEx – Laboratory of Metabolism and Exercise, Faculty of Sport Sciences, University of Porto, Porto, Portugal

**Keywords:** skeletal muscle, histomorphology, intermittent hypobaric hypoxia, capillarisation, VEGF, exercise, simulated altitude

## Abstract

Altitude training has become increasingly popular in recent decades. Its central and peripheral effects are well-described; however, few studies have analyzed the effects of intermittent hypobaric hypoxia (IHH) alone on skeletal muscle morphofunctionality. Here, we studied the effects of IHH on different myofiber morphofunctional parameters, investigating whether contractile activity is required to elicit hypoxia-induced adaptations in trained rats. Eighteen male Sprague-Dawley rats were trained 1 month and then divided into three groups: (1) rats in normobaria (trained normobaric inactive, TNI); (2) rats subjected daily to a 4-h exposure to hypobaric hypoxia equivalent to 4,000 m (trained hypobaric inactive, THI); and (3) rats subjected daily to a 4-h exposure to hypobaric hypoxia just before performing light exercise (trained hypobaric active, THA). After 2 weeks, the tibialis anterior muscle (TA) was excised. Muscle cross-sections were stained for: (1) succinate dehydrogenase to identify oxidative metabolism; (2) myosin-ATPase to identify slow- and fast-twitch fibers; and (3) endothelial-ATPase to stain capillaries. Fibers were classified as slow oxidative (SO), fast oxidative glycolytic (FOG), fast intermediate glycolytic (FIG) or fast glycolytic (FG) and the following parameters were measured: fiber cross-sectional area (FCSA), number of capillaries per fiber (NCF), NCF per 1,000 μm^2^ of FCSA (CCA), fiber and capillary density (FD and CD), and the ratio between CD and FD (C/F). THI rats did not exhibit significant changes in most of the parameters, while THA animals showed reduced fiber size. Compared to TNI rats, FOG fibers from the lateral/medial fields, as well as FIG and FG fibers from the lateral region, had smaller FCSA in THA rats. Moreover, THA rats had increased NCF in FG fibers from all fields, in medial and posterior FIG fibers and in posterior FOG fibers. All fiber types from the three analyzed regions (except the posterior FG fibers) displayed a significantly increased CCA ratio compared to TNI rats. Global capillarisation was also increased in lateral and medial fields. Our results show that IHH alone does not induce alterations in the TA muscle. The inclusion of exercise immediately after the tested hypoxic conditions is enough to trigger a morphofunctional response that improves muscle capillarisation.

## Introduction

Intermittent exposure to hypoxia involves a wide range of physiological, pathophysiological and environmental conditions that induce an equally wide spectrum of physiological responses. These range from harmful maladaptations that can elicit severe health outcomes, such as pulmonary hypertension or fibrosis (Dopp et al., 2007; Schiza et al., 2015), to beneficial adaptations such as improved aerobic capacity and erythropoiesis stimulation (Rodríguez et al., 1999; Navarrete-Opazo and Mitchell, 2014), as well as enhanced cardiac and heart mitochondrial function (Magalhães et al., 2013, 2014). Moreover, intermittent hypoxia (IH) has been proposed or used for several types of non-pharmacological treatments such as therapy for cardiovascular diseases (Serebrovskaya and Xi, 2016) and muscle recovery (Rizo-Roca et al., 2017). Physiological responses to IH are greatly determined by the intensity (altitude), duration and frequency of the exposure to hypoxia (reviewed by Navarrete-Opazo and Mitchell, 2014). Thus, adequate *doses* of IH have been extensively used in recent decades to elicit positive physiological adjustments, especially in altitude acclimation and sport performance that combine exposure to hypoxia with physical exercise (Casas et al., 2000; Vogt and Hoppeler, 2010).

Despite the already well-characterized systemic responses induced by IH (Navarrete-Opazo and Mitchell, 2014), few studies have analyzed in detail the effects of IH on skeletal muscle fiber morphofunctionality. Most are either focused on the effects of chronic hypoxia or on the effects of altitude training. Chronic exposure to hypoxia induces fiber atrophy (Green et al., 1989), loss of mitochondrial density volume (Hoppeler et al., 1990) and increased CD (Deveci et al., 2001), although it remains unclear whether this increase is an indirect consequence of a reduced fiber cross-sectional area (FCSA) (Lundby et al., 2009) or due to angiogenic phenomena, as reported in rodents by Deveci et al. (2002). There is a lack of consensus regarding the effects of altitude training, mainly because of the large differences in the exercise/hypoxia protocols used. Thus, some studies have reported increases in oxidative enzyme levels, myoglobin concentrations, mitochondrial density, FCSA, CD and capillary-to-fiber ratio (C/F) (Desplanches et al., 1993; Geiser et al., 2001; Vogt et al., 2001; Schmutz et al., 2010), while others have reported no changes (Melissa et al., 1997; Masuda et al., 2001) or even decreases in some of these parameters (Bakkman et al., 2007). Previous work from our laboratory has also shown divergent results when exposing rodents to intermittent hypobaric hypoxia (IHH) in daily 4 h at 4,000 m. Panisello et al. (2007) showed IHH-induced increases in myocardial fiber capillarisation associated with a reduced FCSA. Similar results were observed in the diaphragm of the same rats, mainly in slow oxidative (SO) fibers, while no changes were found in the tibialis anterior (TA) muscle, a relatively inactive muscle in resting conditions (Panisello et al., 2008). Thus, we proposed that the effects of IHH strongly depend on the degree of contractile activity of the analyzed muscle: the more active the muscle, the stronger the IHH-induced effects.

In the present study, we aimed to analyse the effects of IHH, alone or in combination with light aerobic exercise (LAE), on the morphofunctional parameters of the TA muscle in trained rats subjected to 2 weeks of a sedentary lifestyle. We hypothesize that LAE immediately after exposure to hypoxia will enhance the response of the TA muscle to hypoxia and be enough to trigger morphofunctional adaptations. Hence, this study could provide further information regarding the use of IHH, alone or in combination with LAE, in the field of altitude medicine, training and acclimatization.

## Materials and Methods

### Animals and Experimental Design

Eighteen male Sprague-Dawley rats were used in this study. All animals had free access to food and water and were maintained at 23°C under a light–dark cycle of 12 h/12 h. Rats were trained twice a day on a treadmill for a month. After this period, the training was stopped and the animals were randomly distributed into three groups (*n* = 6 each): (1) trained animals maintained in normobaric conditions (trained normobaric inactive, TNI); (2) trained animals subjected to daily sessions of hypobaric hypoxia (trained hypobaric inactive, THI); and (3) trained animals subjected to daily sessions of hypobaric hypoxia immediately before a session of LAE on a treadmill (trained hypobaric active, THA). Animals were euthanised 2 weeks after the training period.

All procedures were carried out in accordance with the internal protocols of our laboratory, which were authorized by the University of Barcelona’s Ethical Committee for Animal Experimentation and ratified (file #1899), in accordance with current Spanish legislation, by the *Departament de Medi Ambient i Habitatge* (*Generalitat de Catalunya*).

### Training Protocol

Training sessions were performed twice a day at room temperature (21 ± 2°C) on a motorized treadmill (LE 8710; Panlab, Barcelona, Spain), with a 6-h recovery interval between the sessions. The speed and duration of the training sessions were gradually increased to a speed of 45 cm⋅s^-1^ and a duration of 35 min.

### Intermittent Hypobaric Hypoxia Exposure

Animals were exposed to hypobaric hypoxia for 2 weeks in a hypobaric chamber with a volume of approximately 450 L that provided space for three rat cages. A rotational vacuum pump (TRIVAC D5E; Leybold, Köln, Germany) was used to create a relative vacuum, with its air-flow rate regulated at the inlet by a micrometric valve. Two differential sensors (ID 2000; Leybold, Köln, Germany) driving a diaphragm pressure regulator (MR16; Leybold) controlled the inner pressure of the chamber. The target pressure of 462 torr (equivalent to an altitude of 4,000 m) was achieved steadily over 15 min and maintained for 4 h before restoring normal barometric pressure gradually over 15 min. Animals had free access to food and water inside the hypobaric chamber. Rats belonging to the TNI group were placed over the chamber during the sessions.

### Light Aerobic Exercise

Immediately after exposure to hypobaric hypoxia, THA rats performed LAE to stimulate the contractile activity and aerobic metabolism of their hind limb muscles. This low-impact exercise session consisted of 20 min at 30 cm⋅s^-1^ on a treadmill with an inclination of 5°.

### Muscle Sampling and Histochemical Procedures

Right TA muscles were excised, rinsed in saline solution, immediately frozen in precooled isopentane and stored in liquid nitrogen until analysis.

Muscle samples were embedded in an optimal cutting temperature embedding medium (Tissue-Tek; Sakura Finetek Europe, Zoeterwoude, the Netherlands) at -22°C, and serial transverse sections (12–16 μm) were cut using a cryostat (Leica CM3050S; Wetzlar, Germany). Samples were stained for: (1) endothelial adenosine triphosphatase (eATPase) to reveal muscle capillaries (Fouces et al., 1993); (2) myofibrillar adenosine triphosphatase (mATPase) following alkaline (pH 10.7) pre-incubation to identify slow- and fast-twitch fibers (Brooke and Kaiser, 1970); and (3) succinate dehydrogenase (SDH) to differentiate between aerobic and anaerobic fibers (Nachlas et al., 1957).

### Morphofunctional Measurements

**FIGURE 1:**
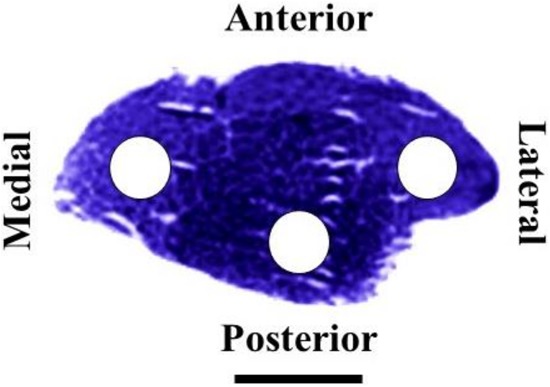
Equatorial transverse section of the right rat tibialis anterior muscle stained for succinate dehydrogenase. Circles indicate lateral, medial and posterior zones selected for histochemical and morphometrical analyses. Scale bar, 5 mm.

Morphofunctional measurements were performed on microphotographs obtained with a light microscope (BX61; Olympus, Tokyo, Japan) connected to a digital camera (DP70; Olympus, Tokyo, Japan) at ×20 magnification. In a previous study conducted in our laboratory (Torrella et al., 2000), we found that rat TA muscle fiber types are unevenly distributed along the muscle (from proximal to distal) and across the muscle cross-section (from lateral to medial and anterior to posterior). Following these previous findings and in order to avoid possible biased results derived from zone selection, we decided to analyse the muscle equatorial zone considering lateral, medial and posterior zones (**Figure 1**). All the parameters listed below were measured or calculated from transverse cross-section tissues with an area of 5.5 × 10^5^ μm^2^ using an image analyzing software (ImageJ; Rasband).

**FIGURE 2:**
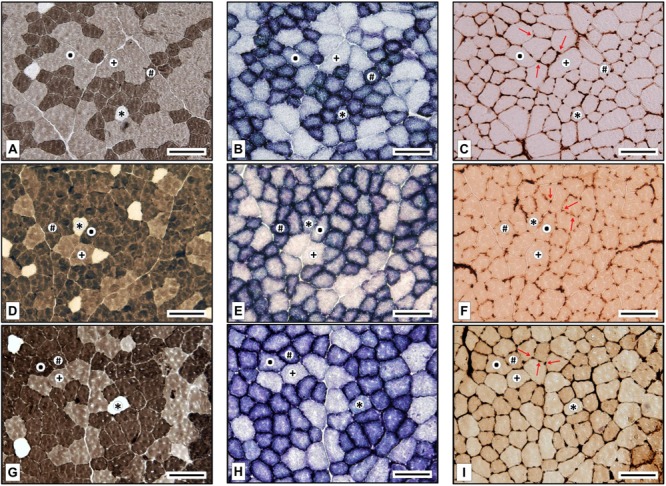
Fiber types in the posterior field of the rat tibialis anterior muscle in Trained Normobaric Inactive **(A–C)**, Trained Hypobaric Inactive **(D–F)**, and Trained Hypobaric Active **(G–I)** animals. **(A,D,G)** Myosin ATPase (alkaline pre-incubation); **(B,E,F)** succinate dehydrogenase; and **(C,F,I)** endothelial ATPase (arrows indicate muscle capillaries). ^∗^, slow oxidative (SO); #, fast oxidative glycolytic (FOG); ∙, fast intermediate glycolytic (FIG); ^†^, fast glycolytic (FG). Scale bar, 100 μm.

All muscle fibers were typified classed as slow-twitch oxidative (SO), fast-twitch oxidative glycolytic (FOG), fast glycolytic (FG), or fast-twitch intermediate glycolytic (FIG). The same researcher performed the fiber typing to ensure the same typing criteria were applied, using software to decide the staining intensity thresholds. **Figure 2** shows representative microphotographs of the eATPase, mATPase, and SDH stainings of all the fiber types identified and for every experimental group. SO fibers had no mATPase activity and displayed high SDH staining; FOG fibers were dark when stained both for mATPase and SDH assays; FG fibers presented moderate mATPase activities and remained unstained after SDH incubation; FIG fibers stained from moderate to high for mATPase and presented an intermediate SDH staining, higher than FG but lower than FOG fibers.

The following parameters were measured or calculated: mean FCSA, circularity (calculated as 4⋅π⋅FCSA/perimeter^2^), number of capillaries per 1,000 μm^2^ of the FCSA (CCA = NCF⋅10^3^/FCSA), number of capillaries per fiber (NCF), FD, CD, and capillary-to-fiber ratio (C/F = CD/FD).

### Citrate Synthase Activity

Citrate synthase activity was assessed as a biomarker of skeletal muscle mitochondrial content. TA muscle was homogenized in ice-cold medium (1:10 w/v) containing 75 mM Tris⋅HCl, 2 mM MgCl_2_, and 1 mM EDTA. The homogenate was centrifuged 5 min at 11,000 × *g* and the supernatant was used to measure the enzymatic activity of the citrate synthase according to Srere (1969).

### VEGF Protein Semiquantification

Thirty milligrams of TA muscle were homogenized in ice-cold lysis buffer (50 mM Tris⋅HCl (pH 8.0), 150 mM NaCl, 1% Triton X-100, 0.5% DOC, and 0.1% SDS) supplemented with protease and phosphate inhibitor cocktails (P8340 and P5726, respectively; Sigma-Aldrich, St. Louis, MO, United States) and centrifuged at 10,000 × *g* for 10 min. The protein concentration of the collected supernatants was determined using the bicinchoninic acid assay (Thermo Scientific, IL, United States). Equivalents amounts of TA muscle protein were separated by SDS/PAGE (10%) and transferred onto PVDF membranes (Millipore, Maine, United States). Membranes were blocked with non-fat dry milk and incubated with anti-VEGF (MA1-16629, Thermo fisher Scientific, United Kingdom) and its correspondent secondary antibody (sc-2005, Santa Cruz, CA, United States). Membranes were photographed using an Odyssey^®^ FC Imaging System (LI-COR Biosciences, NE, United States). Ponceau-S staining was used to normalize differences in protein loading and/or transference.

### Statistical Analysis

Data were analyzed using a one-way ANOVA test followed by the Holm-Sidak *post hoc* test after checking for normality (Kolmogorov-Smirnov test) and homoscedasticity (Levene’s test). The power analysis for the ANOVA test was automatically performed according to the following adjustments: minimum detectable difference in means of 0.8 and alpha of 0.05. A *P* value less than 0.05 was considered statistically significant. The results are reported as mean ± SEM. All statistical tests were performed using SigmaPlot 11 (Systat Software, Inc., 2008–2009).

## Results

### Tibialis Anterior and Animal Body Weight

As shown in **Table 1**, no significant differences were found in the TA and body weights across the different experimental groups, although THA rats had a trend toward lower body weight and higher TA mass. Moreover, when the two parameters were analyzed together, THA animals exhibited higher TA/Body weight ratio than TNI and THI groups (*P* < 0.05).

**Table 1:** Tibialis anterior and body weight.

	TNI	THI	THA
Body weight (g)	473 ± 39	453 ± 39	430 ± 33
Tibialis anterior (g)	1.61 ± 0.21	1.53 ± 0.36	1.72 ± 0.18
Tibialis anterior/Body weight (mg/g)	3.41 ± 0.45	3.23 ± 0.55	3.97 ± 0.32**^∗†^**

*Data are expressed as mean ± standard deviation. Statistically significant differences are indicated as follows: ^∗^*P* < 0.05 vs. TNI; **^†^***P* < 0.05 vs. *TNI*, Trained Normobaric Inactive; *THI*, Trained Hypobaric Inactive; *THA*, Trained Hypobaric Active.*

### Fiber Type Distribution

**FIGURE 3:**
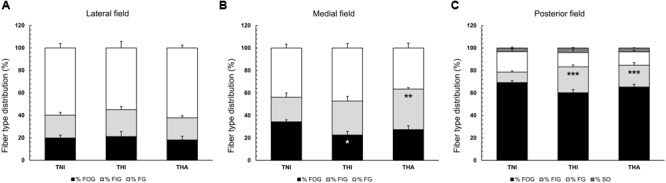
Effects of intermittent hypobaric hypoxia (IHH) alone or in combination with light aerobic exercise on fiber type distribution in three TA muscle regions from trained rats (**A**, lateral; **B**, medial; **C**, posterior). Fibers were classed as fast oxidative glycolytic (FOG), fast intermediate glycolytic (FIG), fast glycolytic (FG), or slow oxidative (SO, only present in the posterior field). ^∗, ∗∗^ and ^∗∗∗^ indicate *P* < 0.05, *P* < 0.01, and *P* < 0.001, respectively, compared to TNI rats. *TNI*, trained normobaric inactive; *THI*, trained hypobaric inactive; *THA*, trained hypobaric active.

Myosin ATPase and SDH staining revealed changes in the fiber type distribution in the TA medial and posterior fields of rats exposed to IHH (**Figure 3**). Thus, THI rats exhibited a decreased percentage of FOG fibers in the medial field compared to TNI animals (22.6 ± 3.3 vs. 34.3 ± 1.8, *P* < 0.05), while a significantly increased percentage of FIG fibers was observed in THA rats compared to TNI animals (36.0 ± 1.3 vs. 21.8 ± 3.8, *P* < 0.01). In the posterior field, both the THI and THA groups showed an increased percentage of FIG fibers compared to normobaric rats (23.0 ± 1.7 and 19.3 ± 2.5 vs. 9.4 ± 1.0, *P* < 0.001).

### Fiber Cross-Sectional Area and Fiber Circularity

**FIGURE 4:**
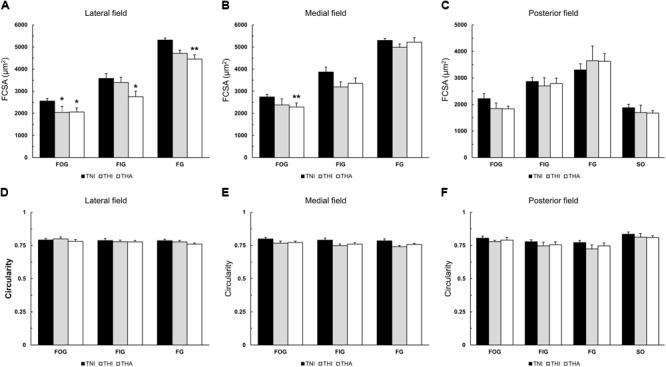
Effects of intermittent hypobaric hypoxia (IHH) alone or in combination with light aerobic exercise on the mean fiber cross-sectional area **(A–C)** and fiber shape **(D–F)** in three TA muscle regions from trained rats. ^∗^ and ^∗∗^ indicate *P* < 0.05 and *P* < 0.01, respectively, compared to TNI rats. *TNI*, trained normobaric inactive; *THI*, trained hypobaric inactive; *THA*, trained hypobaric active.

When combined with LAE, IHH exposure reduced the FCSA in all fiber types of the lateral field (THA: FOG, 2,062 ± 95 μm^2^; FIG, 2,751 ± 151 μm^2^; and FG, 4,452 ± 196 μm^2^ vs. TNI: FOG, 2,556 ± 122 μm^2^; FIG, 3,580 ± 274 μm^2^; and FG, 5,313 ± 184 μm^2^; *P* < 0.05, *P* < 0.05, and *P* < 0.01, respectively) (**Figures 4A–C**). While a similar trend was observed in the other regions of the TA muscle, only FOG fibers from the medial field exhibited a significantly reduced FCSA compared to TNI rats (2,285 ± 126 μm^2^ vs. 2,742 ± 97 μm^2^, respectively, *P* < 0.01). IHH alone (THI) induced a statistically significant decrease only in the lateral FOG fibers (2,047 ± 218 μm^2^, *P* < 0.05).

As shown in **Figures 4D–F**, no differences were observed in fiber circularity, with mean values ranging from 0.77 to 0.81.

### Fiber Capillarisation

**FIGURE 5:**
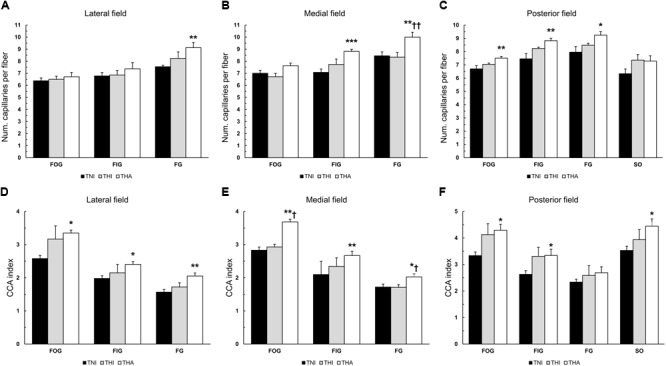
Effects of intermittent hypobaric hypoxia (IHH) alone or in combination with light aerobic exercise on the NCF **(A–C)** and fiber capillarisation index (CCA) **(D–F)** in three TA muscle regions from trained rats. ^∗, ∗∗^, and ^∗∗∗^ indicate *P* < 0.05, *P* < 0.01 and *P* < 0.001, respectively, compared to TNI rats. ^†^ and ^††^ indicate *P* < 0.05 and *P* < 0.01, respectively, compared to THI animals. *TNI*, trained normobaric inactive; *THI*, trained hypobaric inactive; *THA*, trained hypobaric active.

Individual fiber capillarisation was assessed by counting the NCF and calculating the CCA ratio. As can be observed in **Figures 5A–C**, IHH tended to increase the NCF in different fiber types across the three analyzed fields, although without reaching statistical significance. However, when IHH was immediately followed by LAE, this trend was highly accentuated. Compared to the TNI group, THA rats exhibited an increased number of capillaries surrounding the FG fibers in all fields (lateral, 9.1 ± 0.43 vs. 7.5 ± 0.12, *P* < 0.01; medial, 10.0 ± 0.41 vs. 8.5 ± 0.32, *P* < 0.01; and posterior, 9.2 ± 0.27 vs. 8.0 ± 0.42, *P* < 0.05), as well as a higher NCF in the medial and posterior FIG fibers (8.8 ± 0.17 vs. 7.1 ± 0.28, *P* < 0.001, and 8.8 ± 0.18 vs. 7.5 ± 0.40, *P* < 0.01) and posterior FOG fibers (7.5 ± 0.12 vs. 6.7 ± 0.25, *P* < 0.01). For medial FG fibers, THA animals exhibited a higher NCF than THI rats (10 ± 0.41 vs. 8.34 ± 0.41, *P* < 0.01).

Since the NCF strongly depends on fiber size, we also calculated the CCA index, which normalizes the NCF to the FCSA (**Figures 5D–F**). IHH induced a non-significant increase in the CCA ratio in most fiber types across the different regions, which became statistically significant when combined with LAE. Specifically, THA rats had a higher CCA ratio in all the fibers from all fields, with the sole exception of posterior FG fibers.

### Capillary Density and Capillary-to-Fiber Ratio

**FIGURE 6:**
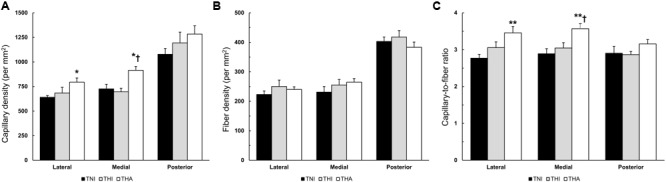
Effects of IHH and IHH+LAE on the **(A)** CD, **(B)** FD, and **(C)** C/F ratio in three TA muscle regions from trained rats. ^∗^ and ^∗∗^ indicate *P* < 0.05 and *P* < 0.01, respectively, compared to TNI rats, while ^†^ indicates *P* < 0.05 compared to THI rats.

Although IHH alone or in combination with LAE did not alter the FD of the TA muscle, IHH together with LAE did increase the CD in all fields. As can be observed in **Figure 6A**, the THA group exhibited a higher CD than TNI animals in the lateral (795 ± 42 vs. 641 ± 17, *P* < 0.05), medial (915 ± 40 vs. 726 ± 47, *P* < 0.05) and posterior fields (1,284 ± 85 vs. 1,078 ± 60, *P* = 0.08), as well as higher medial CD than the THI group (915 ± 40 vs. 697 ± 36, *P* < 0.05). Therefore, as the FD remained unaltered (**Figure 6B**), statistically significant differences between THA and TNI rats were also reported in the C/F ratio in the lateral (3.46 ± 0.17 vs. 2.77 ± 0.10, *P* < 0.01) and medial regions (3.57 ± 0.14 vs. 2.89 ± 0.14, *P* < 0.01) (**Figure 6C**). Statistically significant differences in C/F between the THA and THI rats were only observed in the medial field (3.57 ± 0.14 vs. 3.04 ± 0.14, *P* < 0.05).

### Citrate Synthase Activity

**FIGURE 7:**
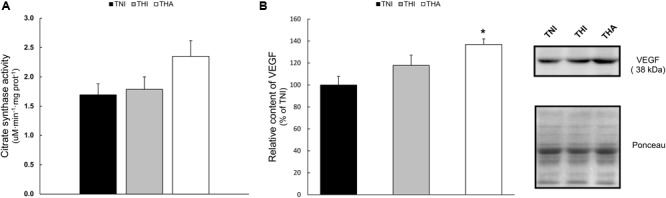
Effects of IHH and IHH+LAE on **(A)** the biomarker of mitochondrial content citrate synthase activity and **(B)** the relative content of the pro-angiogenic factor VEGF in TA muscle homogenates. Band’s intensity were normalized to the Ponceau staining and then relativized to the TNI group. ^∗^ indicates *P* < 0.05 compared to TNI rats.

No statistical differences were found among the different groups in the citrate synthase activity (**Figure 7A**). However, a clear trend was observed in the THA group, in which the activity of this enzyme was 40% higher than in TNI animals (1.69 ± 0.21 vs. 2.35 ± 0.24, *P* = 0.09).

### VEGF Protein Expression

**Figure 7B** shows the semiquantification of the pro-angiogenic protein VEGF by Western Blot. The combination of IHH and LAE (THA rats) induced a significant increase of VEGF expression in TA muscle when compared to the TNI group (THA, 137% ± 11.4, *P* < 0.05).

## Discussion

The effects of chronic hypoxia and altitude training on skeletal muscle morphofunctionality have been extensively analyzed in the last decades (Terrados et al., 1988; Hoppeler et al., 2008; Lundby et al., 2009). However, despite the use of IH in some altitude training protocols, few studies have analyzed its effects (without exercise) on skeletal muscle (Panisello et al., 2008). Here, we provide evidence for the first time that IHH requires a minimum amount of physical exercise to induce significant morphofunctional changes in the TA muscle of trained rats, namely reduced FCSA and increased fiber and muscle capillarization.

### Fiber Type Proportion

Non-uniform distribution of fiber types, as well as variations in their size and capillarisation, is commonly observed in heterogeneous muscles (Bruce and Turek, 1985; Torrella et al., 2000) to enable complex locomotor tasks and adaptation to different metabolic and biomechanical requirements (Gonyea and Ericson, 1977). Consequently, hypoxia-induced morphofunctional alterations have been found to be heterogeneous across different TA muscle regions and fiber types (Deveci et al., 2002; Panisello et al., 2008), highlighting the importance of a detailed and careful analysis when working on histological cross-sections. Thus, THA animals exhibited a significantly increased percentage of FIG fibers in the medial and posterior fields, but not in the lateral region, while THI rats only had more FIG fibers in the posterior region. Surprisingly, this increase was at the expense of both aerobic (FOG) and anaerobic (FG) fibers. Therefore, as FIG fibers exhibit an intermediate oxidative and contractile phenotype, the observed changes could be interpreted as a sign of metabolic flexibility and a higher capacity to adapt to both hypoxic and normoxic conditions. Indeed, the measurement of the citrate synthase activity revealed that the oxidative capacity of the TA muscle of THA rats was not only not compromised despite the decrease of FOG fibers but even displayed non-significant higher enzymatic activity than TNI animals, suggesting that the overall oxidative capacity of the muscle could be improved.

### Fiber Morphometry and Individual Fiber Capillarisation

Despite the lack of changes in fiber type proportion of the lateral field, this region underwent the most pronounced alterations fiber size wise. Thus, IHH followed by LAE reduced the FCSA of all fiber types within the TA lateral region and of the medial FOG fibers, with none of these changes being statistically significant in the posterior area. Interestingly, this region exhibits a smaller mean FCSA and better capillarisation regardless of the fiber type (Torrella et al., 2000), suggesting that hypoxia-induced fiber size adaptations preferentially occur in regions with larger fibers rather than in areas with smaller fibers. Furthermore, as reflected by the different capillary parameters (such as CCA and CD), the lateral and medial fields are irrigated by blood vessels to a lower degree than the posterior region, which could increase their sensitivity to hypoxia. Although chronic hypoxia is usually associated with fiber atrophy (Green et al., 1989; Hoppeler et al., 1990), a reduced FCSA contributes to an overall better irrigation of the muscle and its fibers, allowing more capillaries per mm^2^ and decreasing the oxygen diffusion distance (León-Velarde et al., 1993). Indeed, THA rats did not exhibit an increased NCF of the lateral and medial aerobic fibers (FOG and FIG), but their CCA ratio was found to be increased in all the fields. This ratio normalizes the NCF to the FCSA, thus indicating that fiber size reduction was the main contributor to the increased capillarisation. The results for the FG fibers were particularly interesting: despite a significantly smaller FCSA (lateral field) when compared to TNI animals or an unaltered FCSA (medial and posterior fields), the NCF was higher, thus increasing the CCA ratio. Therefore, the improved irrigation of the FG fibers was due to an increased number of capillaries rather than a consequence of fiber size alteration, suggesting fiber type-specific neovascularization.

So far, these results demonstrate that different muscle regions and fiber types respond distinctly to the hypoxic stimulus, although all tend toward increasing fiber vascularization by means of reduced FCSA, increased NCF or a combination of both. This increased fiber irrigation will facilitate oxygen and energetic substrates supply.

### Global Field Capillarisation

Additionally, our analysis of global field parameters, namely CD, FD, and the C/F ratio, supported previous results. Either because of the FCSA decrease or the NCF increase observed in the different fiber types, the number of capillaries per mm^2^ (i.e., CD) was increased in all TA regions of THA rats (although non-significantly in the posterior) compared to normobaric rats. Similar results were found for the C/F ratio, suggesting an enhanced capillary network occurring through an angiogenic process within the muscle (Hudlicka et al., 1992). This observation was further confirmed by analysing the expression of VEGF, a well-known hypoxia-inducible pro-angiogenic protein (Breen et al., 2008). Animals exposed to IHH+LAE exhibited higher VEGF expression than TNI rats, supporting the histological findings. Furthermore, the C/F ratio has been reported to positively correlate with mitochondrial volume (Poole and Mathieu-Costello, 1996), which would lead to an improved oxidative capacity. Indeed, the analysis of the citrate synthase activity, a biomarker of mitochondrial content, pointed to similar conclusions, as THA animals exhibited a clear increasing trend. Previous studies in humans subjected to hypoxia and exercise also showed increased muscle capillarisation (Desplanches et al., 1993; Schmutz et al., 2010), although Vogt et al. (2001) found increased VEGF expression and capillary length density in the *vastus lateralis* of subjects performing high-intensity altitude training, but not in those performing low-intensity altitude training. However, it should be noted that (in addition to the different experimental model) Vogt et al. (2001) did not carry out region- or fiber type-specific analysis, which could have masked significant changes.

Overall, myofibers from THI rats displayed an intermediate morphological phenotype between those of the TNI and THA animals. This was especially evident in the parameters involving both fiber size and capillarisation, such as the CCA and C/F ratios, where the THI group showed a clear but non-significant increasing trend. Thus, our results suggest that IHH alone does not induce significant fiber morphofunctional adaptations in the TA muscle of trained rats. However, a light exercise protocol in a treadmill immediately after exposure to hypoxia can trigger a wide range of histomorphological alterations. These findings demonstrate that a minimum amount of contractile and metabolic activity is required for a hypoxic response in the TA, probably through further challenging the oxygen homeostasis of the muscle. It is unlikely that the LAE protocol alone could have elicited a similar response in the normobaric trained rats since its intensity, frequency and duration were much lower than the exercise protocol used for the initial training. The initial training protocol involved 35 min on the treadmill at 45 cm⋅s^-1^ twice a day (giving a daily run distance of 1,890 m), while the LAE protocol involved only 20 min at 30 cm⋅s^-1^ (giving a daily run distance of 360 m), which is hardly equivalent to 50% $\dot{\text{V}}$_O_2__max (Powers et al., 1992; Lawler et al., 1993). As has been reviewed elsewhere (Mujika and Padilla, 2000), although trained subjects can preserve, at least partly, muscle adaptations to physical exercise even with reduced regimes, maintaining the training intensity is essential. Furthermore, unpublished data from our laboratory revealed that endurance training led from 10 to 38% increases in FCSA in all fiber types from all TA muscle regions. Thus, if LAE were a stimulus strong enough to induce muscle adaptations *per se* it would be expected to find larger FCSA, contrary to what was observed in THA rats.

## Conclusion

Our results demonstrate that the TA muscle histomorphology of previously trained rats is relatively irresponsive to a daily 4-h exposure to hypobaric hypoxia, consistent with the data of previous studies carried out in sedentary animals (Panisello et al., 2008). However, LAE immediately after exposure to hypoxia is enough to induce changes in fiber size and muscle capillarization in different TA regions. Furthermore, these results open the door to future treatments of musculoskeletal disorders or injury recovery programs in which high-intensity exercise protocols are not recommended.

## Author Contributions

DR-R, TP, GV, and JT conceived and designed the research. DR-R, BÍ, JR-K, and JT conducted sample collection, processing, and data collection. DR-R, JB, and BÍ performed image analysis. DR-R composed the initial manuscript. TP, GV, and JT revised and approved the final draft.

## Conflict of Interest Statement

The authors declare that the research was conducted in the absence of any commercial or financial relationships that could be construed as a potential conflict of interest.

## References

[B1] Bakkman L. Sahlin K. Holmberg H. C. Tonkonogi M. (2007). Quantitative and qualitative adaptation of human skeletal muscle mitochondria to hypoxic compared with normoxic training at the same relative work rate. *Acta Physiol.* 190 243–251. 10.1111/j.1748-1716.2007.01683.x 17521315

[B2] Breen E. Tang K. Olfert M. Knapp A. Wagner P. (2008). Skeletal muscle capillarity during hypoxia: VEGF and its activation. *High Alt. Med. Biol.* 9 158–166. 10.1089/ham.2008.1010 18578647

[B3] Brooke M. H. Kaiser K. K. (1970). Muscle fiber types: how many and what kind? *Arch. Neurol.* 23 369–379. 10.1001/archneur.1970.00480280083010 4248905

[B4] Bruce V. Turek R. J. (1985). Muscle fibre variation in the gluteus medius of the horse. *Equine Vet. J.* 17 317–321. 10.1111/j.2042-3306.1985.tb02508.x 2934247

[B5] Casas M. Casas H. Pagés T. Rama R. Ricart A. Ventura J. L. (2000). Intermittent hypobaric hypoxia induces altitude acclimation and improves the lactate threshold. *Aviat. Space Environ. Med.* 71 125–130. 10685585

[B6] Desplanches D. Hoppeler H. Linossier M. T. Denis C. Claassen H. Dormois D. (1993). Effects of training in normoxia and normobaric hypoxia on human muscle ultrastructure. *Pflügers Arch. Eur. J. Physiol.* 425 263–267. 10.1007/BF00374176 8309787

[B7] Deveci D. Marshall J. M. Egginton S. (2001). Relationship between capillary angiogenesis, fiber type, and fiber size in chronic systemic hypoxia. *Am. J. Physiol. Heart Circ. Physiol.* 281 H241–H252. 10.1007/bf02253829 11406491

[B8] Deveci D. Marshall J. M. Egginton S. (2002). Chronic hypoxia induces prolonged angiogenesis in skeletal muscles of rat. *Exp. Physiol.* 87 287–291. 10.1113/eph8702377 12089595

[B9] Dopp J. M. Reichmuth K. J. Morgan B. J. (2007). Obstructive sleep apnea and hypertension: Mechanisms, evaluation, and management. *Curr. Hypertens. Rep.* 9 529–534. 10.1007/s11906-007-0095-2 18367017

[B10] Fouces V. Torrella J. R. Palomeque J. Viscor G. (1993). A histochemical ATPase method for the demonstration of the muscle capillary network. *J. Histochem. Cytochem.* 41 283–289. 10.1177/41.2.7678272 7678272

[B11] Geiser J. Vogt M. Billeter R. Zuleger C. Belforti F. Hoppeler H. (2001). Training high - living low: changes of aerobic performance and muscle structure with training at simulated altitude. *Int. J. Sports Med.* 22 579–585. 10.1055/s-2001-18521 11719893

[B12] Gonyea W. J. Ericson G. C. (1977). Morphological and histochemical organization of the flexor carpi radialis muscle in the cat. *Am. J. Anat.* 148 329–344. 10.1002/aja.1001480304 193389

[B13] Green H. J. Sutton J. R. Cymerman A. Young P. M. Houston C. S. (1989). Operation Everest II: adaptations in human skeletal muscle. *J. Appl. Physiol.* 66 2454–2461. 10.1152/jappl.1989.66.5.2454 2745306

[B14] Hoppeler H. Kleinert E. Schlegel C. Claassen H. Howald H. Kayar S. R. (1990). Morphological adaptations of human skeletal muscle to chronic hypoxia. *Int. J. Sports Med.* 11(Suppl. 1) S3–S9. 10.1055/s-2007-1024846 2323861

[B15] Hoppeler H. Klossner S. Vogt M. (2008). Training in hypoxia and its effects on skeletal muscle tissue. *Scand. J. Med. Sci. Sports* 18(Suppl. 1) 38–49. 10.1111/j.1600-0838.2008.00831.x 18665951

[B16] Hudlicka O. Brown M. Egginton S. (1992). Angiogenesis in skeletal and cardiac muscle. *Physiol. Rev.* 72 369–400. 10.1152/physrev.1992.72.2.369 1372998

[B17] Lawler J. M. Powers S. K. Hammeren J. Martin A. D. (1993). Oxygen cost of treadmill running in 24-month-old Fischer 344 rats. *Med. Sci. Sports Exerc.* 25 1259–1264. 10.1249/00005768-199311000-00009 8289613

[B18] León-Velarde F. Sanchez J. Bigard A. X. Brunet A. Lesty C. Monge C. (1993). High altitude tissue adaptation in Andean coots: capillarity, fibre area, fibre type and enzymatic activities of skeletal muscle. *J. Comp. Physiol. B* 163 52–58. 10.1007/BF00309665 8459054

[B19] Lundby C. Calbet J. A. L. Robach P. (2009). The response of human skeletal muscle tissue to hypoxia. *Cell Mol. Life. Sci.* 66 3615–3623. 10.1007/s00018-009-0146-8 19756383 PMC11115669

[B20] Magalhães J. Falcão-Pires I. Gonçalves I. O. Lumini-Oliveira J. Marques-Aleixo I. Dos Passos E. (2013). Synergistic impact of endurance training and intermittent hypobaric hypoxia on cardiac function and mitochondrial energetic and signaling. *Int. J. Cardiol.* 168 5363–5371. 10.1016/j.ijcard.2013.08.001 24012275

[B21] Magalhães J. Gonçalves I. O. Lumini-Oliveira J. Marques-Aleixo I. Passos E. Rocha-Rodrigues S. (2014). Modulation of cardiac mitochondrial permeability transition and apoptotic signaling by endurance training and intermittent hypobaric hypoxia. *Int. J. Cardiol.* 173 40–45. 10.1016/j.ijcard.2014.02.011 24602319

[B22] Masuda K. Okazaki K. Kuno S. Asano K. Shimojo H. Katsuta S. (2001). Endurance training under 2500-m hypoxia does not increase myoglobin content in human skeletal muscle. *Eur. J. Appl. Physiol.* 85 486–490. 10.1007/s004210100471 11606019

[B23] Melissa L. MacDougall J. D. Tarnopolsky M. A. Cipriano N. Green H. J. (1997). Skeletal muscle adaptations to training under normobaric hypoxic versus normoxic conditions. *Med. Sci. Sport Exerc.* 29 238–243. 10.1097/00005768-199702000-00012 9044229

[B24] Mujika I. Padilla S. (2000). Detraining: loss of training-induced physiological and performance adaptations. Part II: long term insufficient training stimulus. *Sports Med.* 30 145–154. 10.2165/00007256-200030030-00001 10999420

[B25] Nachlas M. M. Tsou K. C. De Souza E. Cheng C. S. Seligman A. M. (1957). Cytochemical demonstration of succinic dehydrogenase by the use of a new p-nitrophenyl substituted ditetrazole. *J. Histochem. Cytochem.* 5 420–436. 10.1177/5.4.420 13463314

[B26] Navarrete-Opazo A. Mitchell G. S. (2014). Therapeutic potential of intermittent hypoxia: a matter of dose. *Am. J. Physiol. Regul. Integr. Comp. Physiol.* 307 R1181–R1197. 10.1152/ajpregu.00208.2014 25231353 PMC4315448

[B27] Panisello P. Torrella J. R. Esteva S. Pagés T. Viscor G. (2008). Capillary supply, fibre types and fibre morphometry in rat tibialis anterior and diaphragm muscles after intermittent exposure to hypobaric hypoxia. *Eur. J. Appl. Physiol.* 103 203–213. 10.1007/s00421-008-0691-0 18270729

[B28] Panisello P. Torrella J. R. Pagés T. Viscor G. (2007). Capillary supply and fiber morphometry in rat myocardium after intermittent exposure to hypobaric hypoxia. *High Alt. Med. Biol.* 8 322–330. 10.1089/ham.2007.1030 18081508

[B29] Poole D. C. Mathieu-Costello O. (1996). Relationship between fiber capillarization and mitochondrial volume density in control and trained rat soleus and plantaris muscles. *Microcirculation* 3 175–186. 10.3109/10739689609148286 8839439

[B30] Powers S. K. Lawler J. Criswell D. Lieu F. K. Martin D. (1992). Ageing and respiratory muscle metabolic plasticity: effects of endurance training. *J. Appl. Physiol.* 72 1068–1073. 10.1152/jappl.1992.72.3.1068 1568962

[B31] Rizo-Roca D. Ríos-Kristjánsson J. G. Núñez-Espinosa C. Santos-Alves E. Gonçalves I. O. Magalhães J. (2017). Intermittent hypobaric hypoxia combined with aerobic exercise improves muscle morphofunctional recovery after eccentric exercise to exhaustion in trained rats. *J. Appl. Physiol.* 122 580–592. 10.1152/japplphysiol.00501.2016 27765844

[B32] Rodríguez F. A. Casas H. Casas M. Pagés T. Rama R. Ricart A. (1999). Intermittent hypobaric hypoxia stimulates erythropoiesis and improves aerobic capacity. *Med. Sci. Sports Exerc.* 31 264–268. 10.1097/00005768-199902000-00010 10063816

[B33] Schiza S. Mermigkis C. Margaritopoulos G. A. Daniil Z. Harari S. Poletti V. (2015). Idiopathic pulmonary fibrosis and sleep disorders: no longer strangers in the night. *Eur. Respir. Rev.* 24 327–339. 10.1183/16000617.00009114 26028644 PMC9487812

[B34] Schmutz S. Däpp C. Wittwer M. Durieux A. C. Mueller M. Weinstein F. (2010). A hypoxia complement differentiates the muscle response to endurance exercise. *Exp. Physiol.* 95 723–735. 10.1113/expphysiol.2009.051029 20176680

[B35] Serebrovskaya T. V. Xi L. (2016). Intermittent hypoxia training as non-pharmacologic therapy for cardiovascular diseases: practical analysis on methods and equipment. *Exp. Biol. Med. (Maywood)* 241 1708–1723. 10.1177/1535370216657614 27407098 PMC4999622

[B36] Srere P. A. (1969). [1] Citrate synthase: [EC 4.1.3.7. Citrate oxaloacetate-lyase (CoA-acetylating)]. *Methods Enzymol.* 13 3–11. 10.1016/0076-6879(69)13005-0

[B37] Terrados N. Melichna J. Sylvén C. Jansson E. Kaijser L. (1988). Effects of training at simulated altitude on performance and muscle metabolic capacity in competitive road cyclists. *Eur. J. Appl. Physiol. Occup. Physiol.* 57 203–209. 10.1007/BF00640664 3349988

[B38] Torrella J. R. Whitmore J. M. Casas M. Fouces V. Viscor G. (2000). Capillarity, fibre types and fibre morphometry in different sampling sites across and along the tibialis anterior muscle of the rat. *Cells Tissues Organs* 167 153–162. 10.1159/000016778 10971039

[B39] Vogt M. Hoppeler H. (2010). Is hypoxia training good for muscles and exercise performance? *Prog. Cardiovasc. Dis.* 52 525–533. 10.1016/j.pcad.2010.02.013 20417346

[B40] Vogt M. Puntschart A. Geiser J. Zuleger C. Billeter R. Hoppeler H. (2001). Molecular adaptations in human skeletal muscle to endurance training under simulated hypoxic conditions. *J. Appl. Physiol.* 91 173–182. 10.1152/jappl.2001.91.1.173 11408428

